# Viruses and Bats

**DOI:** 10.3390/v11100884

**Published:** 2019-09-21

**Authors:** Patrick C. Y. Woo, Susanna K. P. Lau

**Affiliations:** 1 Department of Microbiology, The University of Hong Kong, Hong Kong, China; 2 State Key Laboratory of Emerging Infectious Diseases, The University of Hong Kong, Hong Kong, China; 3 Collaborative Innovation Center for Diagnosis and Treatment of Infectious Diseases, The University of Hong Kong, Hong Kong, China

Bats are a unique but diverse group of mammals in the order *Chiroptera* (meaning “hand-wings” in Greek), which is the second largest order of mammals in terms of species number. Thus far, there are more than 1300 known species of bats across six continents, constituting 20% of the total number of mammalian species. Indonesia, for example, is home of more than 200 bat species, the largest variety in the world. Bats are monophyletic. Traditionally, they were classified into two suborders: *Megachiroptera* (megabats) and *Microchiroptera* (microbats). In general, megabats are larger than microbats, although some large microbats can be larger than some small megabats. Most megabats are frugivorous and eat fruit, nectar or pollen and most microbats are insectivorous. Some microbats also eat small vertebrates or even other bats. Recently, extensive molecular studies and phylogenetic analysis have led to a revision of the classification of bats into two suborders, *Yinpterochiroptera* and *Yangochiroptera*, of which *Yinpterochiroptera* includes the megabats and a number of microbat species. In general, bats are nocturnal animals that are mainly active during night time, dusk or dawn. They roost in caves, trees, mines and water tunnels and most rest and sleep in an upside-down position. They usually live in groups known as colonies, with hundreds to thousands of individuals, which may facilitate intraspecies transmission of viruses. They are capable of echolocation, which help them to locate themselves as well as their food. 

The high diversity of bats, and hence the highly diversified cell types and receptors, have facilitated this group of animals to harbor a myriad of viruses. Bats are well recognized to be the hosts of a number of highly pathogenic viruses, such as rabies virus, Hendra virus, Nipah virus and Ebola virus, for a long time. Since the discovery of bats as the reservoir of Severe Acute Respiratory Syndrome (SARS) coronavirus [1,2], there has been a surge of interests in research on various aspects of bat viruses as well as bat-virus interactions. Using coronavirus as an example, there have been a total of at least 30 bat coronaviruses discovered in the last 15 years after the SARS epidemic in 2003 [3,4,5,6,7,8,9,10,11,12,13,14,15], and bats are now recognized as the gene source of *Alphacoronavirus* and *Betacoronavirus* in the current model of coronavirus evolution, the only two coronavirus genera with coronaviruses that are known to infect humans [16]. The discovery of *Tylonycteris* bat CoV HKU4 and *Pipistrellus* bat CoV HKU5 in *Tylonycteris pachypus* and *Pipistrellus abramus,* respectively, have also greatly facilitated the study of the Middle East Respiratory Syndrome coronavirus when it emerged in the Middle East in 2012 [9,17]. In fact, among the ~1900 papers on [bat virus] indexed in PubMed, two-thirds of them were published after the SARS epidemic in 2003 and half of them were published in the last seven years. In 2013, bats were the known reservoirs of more than 60 viruses that can infect humans. 

In addition to its diversity, the capability to fly also facilitate bats to disseminate the viruses they harbor, and hence, increases the chance of both intraspecies and interspecies transmission, which involves two bat species or one bat species and another kind of animal. With their forelimbs adapted as wings, bats are the only mammals that are capable of sustained flight. Echolocation enables bats to fly in dark places. Bats have long finger-like bones in their wings, and hence are more maneuverable than the wings of birds. However, unlike birds, they do not have a lot of lift. Furthermore, the fact that they are nocturnally active helps them avoid direct competition with birds. The ability to fly makes bats important vehicles for the dissemination of seeds and pollens, as well as viruses. 

Contacts between human and bats or bat products are not uncommon. Huge bat caves, such as the Batu Caves in Malaysia, are major tourist attractions. Caves explorers are sometimes bitten by bats, and their mucous membranes or wounds may contact the saliva, urine, etc. of bats, resulting in virus transmission. In addition to these direct contacts, bats are used as food in southern China and some parts of Asia such as Indonesia. A variety of bat dishes, minced bat meat, and even hot pot with the whole bat cooked in a pot of soup are available in restaurants in southern China. Dried bat droppings are used as traditional Chinese medicine, for the treatment of diseases such as night blindness. Bat dung that is mined in caves as guano can be used as organic fertilizers. All these uses of bats and their derived products have created countless opportunities of human-bat interaction, and hence, have increased the chance of virus transmission. 

In the recent few years, numerous novel bat viruses have been discovered and other downstream studies performed. In this special issue, a number of novel bat viruses, including coronavirus, poxvirus, paramyxovirus and orbivirus, were described [18,19,20,21,22,23,24]. In addition, molecular surveillance studies have improved our understanding on the epidemiology of a number of virus families in bats, as for example, adenovirus, coronavirus, mobatvirus, rubulavirus and rotavirus [15,25,26,27,28,29,30,31]. Furthermore, a number of studies on bat-virus interaction have provided insights into the innate immune response of bats developed upon encountering bat viruses [32,33,34,35,36,37,38,39]. All these have helped us better understand the diversity, epidemiology, intraspecies and interspecies transmission, host-virus interaction, etc. in various virus families in this unique group of animals. 

## References

[1] Lau S.K., Woo P.C., Li K.S., Huang Y., Tsoi H.W., Wong B.H., Wong S.S., Leung S.Y., Chan K.H., Yuen K.Y. (2005). Severe acute respiratory syndrome coronavirus-like virus in Chinese horseshoe bats. Proc. Natl. Acad. Sci. USA.

[2] Li W., Shi Z., Yu M., Ren W., Smith C., Epstein J.H., Wang H., Crameri G., Hu Z., Zhang H. (2005). Bats are natural reservoirs of SARS-like coronaviruses. Science.

[3] Corman V.M., Ithete N.L., Richards L.R., Schoeman M.C., Preiser W., Drosten C., Drexler J.F. (2014). Rooting the Phylogenetic Tree of Middle East Respiratory Syndrome Coronavirus by Characterization of a Conspecific Virus from an African Bat. J. Virol..

[4] Lau S.K., Woo P.C., Li K.S., Huang Y., Wang M., Lam C.S., Xu H., Guo R., Chan K.H., Zheng B.J. (2007). Complete genome sequence of bat coronavirus HKU2 from Chinese horseshoe bats revealed a much smaller spike gene with a different evolutionary lineage from the rest of the genome. Virology.

[5] Lau S.K.P., Li K.S.M., Huang Y., Shek C.T., Tse H., Wang M., Choi G.K.Y., Xu H., Lam C.S.F., Guo R. (2010). Ecoepidemiology and Complete Genome Comparison of Different Strains of Severe Acute Respiratory Syndrome-Related Rhinolophus Bat Coronavirus in China Reveal Bats as a Reservoir for Acute, Self-Limiting Infection That Allows Recombination Events. J. Virol..

[6] Lau S.K., Li K.S., Tsang A.K., Shek C.T., Wang M., Choi G.K., Guo R., Wong B.H., Poon R.W., Lam C.S. (2012). Recent transmission of a novel alphacoronavirus, bat coronavirus HKU10, from Leschenault’s rousettes to pomona leaf-nosed bats: First evidence of interspecies transmission of coronavirus between bats of different suborders. J. Virol..

[7] Woo P.C., Lau S.K., Li K.S., Poon R.W., Wong B.H., Tsoi H.W., Yip B.C., Huang Y., Chan K.H., Yuen K.Y. (2006). Molecular diversity of coronaviruses in bats. Virology.

[8] Huang C., Liu W.J., Xu W., Jin T., Zhao Y., Song J., Shi Y., Ji W., Jia H., Zhou Y. (2016). A Bat-Derived Putative Cross-Family Recombinant Coronavirus with a Reovirus Gene. PLoS Pathog.

[9] Woo P.C., Wang M., Lau S.K., Xu H., Poon R.W., Guo R., Wong B.H., Gao K., Tsoi H.W., Huang Y. (2007). Comparative analysis of twelve genomes of three novel group 2c and group 2d coronaviruses reveals unique group and subgroup features. J. Virol..

[10] Anthony S.J., Gilardi K., Menachery V.D., Goldstein T., Ssebide B., Mbabazi R., Navarrete-Macias I., Liang E., Wells H., Hicks A. (2017). Further Evidence for Bats as the Evolutionary Source of Middle East Respiratory Syndrome Coronavirus. mBio.

[11] Yang L., Wu Z., Ren X., Yang F., Zhang J., He G., Dong J., Sun L., Zhu Y., Zhang S. (2014). MERS-related betacoronavirus in Vespertilio superans bats, China. Emerg. Infect. Dis..

[12] Moreno A., Lelli D., De Sabato L., Zaccaria G., Boni A., Sozzi E., Prosperi A., Lavazza A., Cella E., Castrucci M.R. (2018). Detection and full genome characterization of two beta CoV viruses related to Middle East respiratory syndrome from bats in Italy (vol 14, 1, 2017). Virol. J..

[13] Lau S.K.P., Zhang L., Luk H.K.H., Xiong L., Peng X., Li K.S.M., He X., Zhao P.S., Fan R.Y.Y., Wong A.C.P. (2018). Receptor Usage of a Novel Bat Lineage C Betacoronavirus Reveals Evolution of Middle East Respiratory Syndrome-Related Coronavirus Spike Proteins for Human Dipeptidyl Peptidase 4 Binding. J. Infect. Dis..

[14] Luo C.M., Wang N., Yang X.L., Liu H.Z., Zhang W., Li B., Hu B., Peng C., Geng Q.B., Zhu G.J. (2018). Discovery of Novel Bat Coronaviruses in South China That Use the Same Receptor as Middle East Respiratory Syndrome Coronavirus. J. Virol..

[15] Wong A.C.P., Li X., Lau S.K.P., Woo P.C.Y. (2019). Global Epidemiology of Bat Coronaviruses. Viruses.

[16] Woo P.C., Lau S.K., Lam C.S., Lau C.C., Tsang A.K., Lau J.H., Bai R., Teng J.L., Tsang C.C., Wang M. (2012). Discovery of seven novel Mammalian and avian coronaviruses in the genus deltacoronavirus supports bat coronaviruses as the gene source of alphacoronavirus and betacoronavirus and avian coronaviruses as the gene source of gammacoronavirus and deltacoronavirus. J. Virol..

[17] Woo P.C., Lau S.K., Li K.S., Tsang A.K., Yuen K.Y. (2012). Genetic relatedness of the novel human group C betacoronavirus to Tylonycteris bat coronavirus HKU4 and Pipistrellus bat coronavirus HKU5. Emerg. Microbes Infect..

[18] Lelli D., Lavazza A., Prosperi A., Sozzi E., Faccin F., Baioni L., Trogu T., Cavallari G.L., Mauri M., Gibellini A.M. (2019). Hypsugopoxvirus: A Novel Poxvirus Isolated from *Hypsugo savii* in Italy. Viruses.

[19] Lau S.K.P., Wong A.C.P., Zhang L., Luk H.K.H., Kwok J.S.L., Ahmed S.S., Cai J.P., Zhao P.S.H., Teng J.L.L., Tsui S.K.W. (2019). Novel Bat Alphacoronaviruses in Southern China Support Chinese Horseshoe Bats as an Important Reservoir for Potential Novel Coronaviruses. Viruses.

[20] Wang N., Luo C., Liu H., Yang X., Hu B., Zhang W., Li B., Zhu Y., Zhu G., Shen X. (2019). Characterization of a New Member of Alphacoronavirus with Unique Genomic Features in Rhinolophus Bats. Viruses.

[21] Fagre A.C., Lee J.S., Kityo R.M., Bergren N.A., Mossel E.C., Nakayiki T., Nalikka B., Nyakarahuka L., Gilbert A.T., Peterhans J.K. (2019). Discovery and Characterization of Bukakata orbivirus (Reoviridae:Orbivirus), a Novel Virus from a Ugandan Bat. Viruses.

[22] Mendenhall I.H., Kerimbayev A.A., Strochkov V.M., Sultankulova K.T., Kopeyev S.K., Su Y.C.F., Smith G.J.D., Orynbayev M.B. (2019). Discovery and Characterization of Novel Bat Coronavirus Lineages from Kazakhstan. Viruses.

[23] Johnson R.I., Tachedjian M., Rowe B., Clayton B.A., Layton R., Bergfeld J., Wang L.F., Marsh G.A. (2018). Alston Virus, a Novel Paramyxovirus Isolated from Bats Causes Upper Respiratory Tract Infection in Experimentally Challenged Ferrets. Viruses.

[24] Lazov C.M., Chriel M., Baagoe H.J., Fjederholt E., Deng Y., Kooi E.A., Belsham G.J., Botner A., Rasmussen T.B. (2018). Detection and Characterization of Distinct Alphacoronaviruses in Five Different Bat Species in Denmark. Viruses.

[25] Arai S., Kikuchi F., Bawm S., Son N.T., Lin K.S., Tu V.T., Aoki K., Tsuchiya K., Tanaka-Taya K., Morikawa S. (2019). Molecular Phylogeny of Mobatviruses (Hantaviridae) in Myanmar and Vietnam. Viruses.

[26] Diakoudi G., Lanave G., Moreno A., Chiapponi C., Sozzi E., Prosperi A., Larocca V., Losurdo M., Decaro N., Martella V. (2019). Surveillance for Adenoviruses in Bats in Italy. Viruses.

[27] Mendenhall I.H., Wen D.L.H., Jayakumar J., Gunalan V., Wang L., Mauer-Stroh S., Su Y.C.F., Smith G.J.D. (2019). Diversity and Evolution of Viral Pathogen Community in Cave Nectar Bats (*Eonycteris spelaea*). Viruses.

[28] Mortlock M., Dietrich M., Weyer J., Paweska J.T., Markotter W. (2019). Co-Circulation and Excretion Dynamics of Diverse Rubula- and Related Viruses in Egyptian Rousette Bats from South Africa. Viruses.

[29] Phelps K.L., Hamel L., Alhmoud N., Ali S., Bilgin R., Sidamonidze K., Urushadze L., Karesh W., Olival K.J. (2019). Bat Research Networks and Viral Surveillance: Gaps and Opportunities in Western Asia. Viruses.

[30] Fan Y., Zhao K., Shi Z.L., Zhou P. (2019). Bat Coronaviruses in China. Viruses.

[31] Banerjee A., Kulcsar K., Misra V., Frieman M., Mossman K. (2019). Bats and Coronaviruses. Viruses.

[32] Schountz T., Campbell C., Wagner K., Rovnak J., Martellaro C., DeBuysscher B.L., Feldmann H., Prescott J. (2019). Differential Innate Immune Responses Elicited by Nipah Virus and Cedar Virus Correlate with Disparate in vivo Pathogenesis in Hamsters. Viruses.

[33] Laing E.D., Sterling S.L., Weir D.L., Beauregard C.R., Smith I.L., Larsen S.E., Wang L.F., Snow A.L., Schaefer B.C., Broder C.C. (2019). Enhanced Autophagy Contributes to Reduced Viral Infection in Black Flying Fox Cells. Viruses.

[34] Banerjee A., Falzarano D., Rapin N., Lew J., Misra V. (2019). Interferon Regulatory Factor 3-Mediated Signaling Limits Middle-East Respiratory Syndrome (MERS) Coronavirus Propagation in Cells from an Insectivorous Bat. Viruses.

[35] Balkema-Buschmann A., Rissmann M., Kley N., Ulrich R., Eiden M., Groschup M.H. (2018). Productive Propagation of Rift Valley Fever Phlebovirus Vaccine Strain MP-12 in Rousettus aegyptiacus Fruit Bats. Viruses.

[36] Arnold C.E., Guito J.C., Altamura L.A., Lovett S.P., Nagle E.R., Palacios G.F., Sanchez-Lockhart M., Towner J.S. (2018). Transcriptomics Reveal Antiviral Gene Induction in the Egyptian Rousette Bat Is Antagonized In Vitro by Marburg Virus Infection. Viruses.

[37] Kumar N., Kulkarni D.D., Lee B., Kaushik R., Bhatia S., Sood R., Pateriya A.K., Bhat S., Singh V.P. (2018). Evolution of Codon Usage Bias in Henipaviruses Is Governed by Natural Selection and Is Host-Specific. Viruses.

[38] Fagre A.C., Kading R.C. (2019). Can Bats Serve as Reservoirs for Arboviruses?. Viruses.

[39] Subudhi S., Rapin N., Misra V. (2019). Immune System Modulation and Viral Persistence in Bats: Understanding Viral Spillover. Viruses.

